# An Acute Ibuprofen Overdose Masking a Severe *Staphylococcus aureus* Meningitis: A Case Report

**DOI:** 10.1155/2013/603251

**Published:** 2013-06-16

**Authors:** Matthew Smetana, Katy Picard, Kevin M. Boehm

**Affiliations:** ^1^Department of Emergency Medicine, Henry Ford Wyandotte Hospital, Wyandotte, MI 48192, USA; ^2^College of Osteopathic Medicine, A.T. Still University, Kirksville, MO 63501, USA; ^3^College of Osteopathic Medicine, Michigan State University, East Lansing, MI 48824, USA; ^4^College of Osteopathic Medicine, Nova Southeastern University, Davie, FL 33328, USA

## Abstract

Acute bacterial meningitis has a low incidence (3/100,000 in the United States) and yet high fatality rate (approximately 14–16%) and classically presents as a triad of fever, neck stiffness, and altered mental status. However, less than half of patients with meningitis present with this classic triad. We present the clinical course of a patient who initially presented to the emergency department after overdosing on ibuprofen for what he described as back pain secondary to mechanical injury. However, the patient's condition quickly deteriorated: he developed tachycardia, mental status changes, was intubated due to respiratory distress, and then suffered an 8-minute PEA arrest before return of spontaneous circulation was achieved. After the patient was stabilized, in addition to the nonsteroidal anti-inflammatory drug (NSAID) overdose *Staphylococcus aureus* meningitis, bacteremia, and pneumonia were diagnosed. We report this case to illustrate that the initial presentation of meningitis may be extremely unusual especially in the setting of NSAID overdose and the acutely decompensating patient. As the risk of adverse clinical outcomes increases with delays in appropriate antibiotic therapy, it is therefore crucial to recognize the many signs and symptoms of meningitis, typical and atypical, and quickly begin appropriate treatment.

## 1. Introduction

This case exemplifies some of the challenges of diagnosing acute bacterial meningitis in the context of acute ibuprofen overdose. A 53-year-old male presented to a community based academic emergency department by means of a personal vehicle. Additional diagnosis are explored and eventual diagnosis of bacterial meningitis is made. Treatment is started and the patient had a poor clinical outcome secondary to failure of prompt diagnosis. The initial workup and stabilization of acute ibuprofen overdoes are discussed as well as current recommendations for the diagnosis and treatment of bacterial meningitis. 

## 2. Case Presentation

 A 53-year-old male presented to an academic community Emergency department via personal vehicle. The patient states that he had been having increasing low back pain for four days following a twisting injury at work while lifting a heavy object. At time of presentation, the patient was ambulatory with steady gait. The patient states that, while lifting a box of heavy papers from the trunk of his car, he rotated to the right and felt a “pop.” Following this episode he had pain to the point that he states that he found it painful to get out of bed and attempted to relieve his pain with what he described as seventy ibuprofen tablets. The patient described that pain as constant and sharp in nature, aggravated with rotation of his lumbar spine, and relieved with immobilization. At initial triage the patient rated his pain at 10 on a 10-point scale. 

 Prior to this episode the patient described himself as a healthy male denying any past medical, surgical psychiatric, or contributing family history. The patient initially denied any tobacco, alcohol, or ilicit drug use. In the course of the patient interview it was found that the review of systems was positive for general malaise, weakness, diaphoresis, nausea with no emesis, and low back spasms. The review of systems was negative for fever, headache, rash, change in vision, chest pain, dyspnea, cough, hemoptysis, constipation, diarrhea, bowel or bladder incontinence, hematemesis, melena, hematochezia, dysuria, hematuria, saddle anesthesia, or suicidal ideations. 

On physical exam the patient initially appeared in moderate distress as he constantly shifted positions on the stretcher in an attempt to find a comfortable position. The patient was initially alert and orientated to person, place, time, and event with a blood pressure 148/62, pulse 63, respirations 20, oral temperature at 36.5, and pulse oximetry 98% on room air. No trauma was noted to the patient's head neck or back. The patient's pupils were noted to be equal, round and reactive to light, the extraocular movements were normal and the conjunctivas were noted to be normal. Examination of the ears, nose, and throat were noted to have no signs of infection or trauma. There were no meningeal signs noted, however, some pain was elicited with palpation of the cervical paraspinal muscles. The neck did show a normal range of motion and the patient's trachea was midline. The chest exam noted normal bilateral breath sounds with the heart rate noted to be a regular rate and rhythm. The patient's heart sounds were normal. The abdomen was soft, nontender, and nondistended with normal bowel sounds. The patient's rectal exam was normal with normal rectal tone. On examination of this patient's back, it was noted that he had decreased range of motion limited by pain and tenderness to palpation on the midline as well as bilateral paraspinal musculature. Overlying the lumbar spine there were no skin lesions. A straight leg test was positive at 30 degrees. Muscle strength as well as sensation and distal pulses was normal in both the upper and lower extremities. There was no edema noted in the upper or lower extremities. No focal neurological deficits were noted and cranial nerves, two through twelve, were grossly normal as tested. A Glascow Coma Score was 15. Other than being diffusely diaphoretic, the skin was grossly intact. Psychiatric examination was positive for an anxious affect.

 Initial labs were obtained as shown in Table 1 below and were interpreted. The urine drug screen was positive for opiates and benzodiazepines although this was obtained after administration of diazepam and hydrocodone for treatment of his back pain. Initial electrolytes showed a low sodium of 122 mmol/L and an elevated anion gap at 15 with a corresponding lactic acid at 3.6 mmol/L. Initial arterial blood gas showed a partially compensated respiratory alkalosis. Kidney function tests showed an intrinsic renal failure. Urinanalysis was clean with some proteinuria. Initial CBC showed no leukocytosis; however, there was a significant left shift. An initial EKG was nonspecific for any acute abnormality and initial troponin was within normal range. An initial Chest X-ray was read as vascular congestion. 

 Initial differential diagnosis was focused on a known ingestion of ibuprofen with possible additional toxic ingestion. Additional differential diagnoses included renal failure, respiratory distress, myocardial infarction, and finally sepsis. Despite 2 mg of hydrocodone intravenous push (IVP) and 5 mg of diazapam IVP, the patient continued to complain of 10/10 low back pain. Poison control was contacted and oral charcoal and fomepizole were given for suspected NSAID overdose as a nidus for the patients condition. Fomepizole was given for possible toxic alcohol ingestion with elevated anion gap. Over the course of the next hour the patient became very tachypneic (rate of 48 breaths per minute), tachycardic (pulse rate 163 beats per minute), hypoxic (Spo2 90% on 15 liters per minute by nonrebreather mask) hypertensive (205/80) and diaphoretic. The patient was subsequently intubated for respiratory distress without incident. 

The patient was admitted to the intensive care unit. A head CT was obtained due to the patient's change in mental status, which failed to demonstrate any hemorrhage or mass effect. Shortly thereafter, the patient became hypotensive, suffered a pulseless electrical activity (PEA) arrest, and was recovered after approximately 8 minutes of cardiopulmonary resuscitation and the administration of epinephrine, atropine, and bicarbonate. Following cardiac arrest the patienthad a mild global hypokinesis of the left ventricle and an ejection fraction of 40–45% on 2D echocardiography. The patient continued to be tachycardic and was found to have a temperature of 40.6 degrees Celsius, for which cooling blankets were placed upon the patient. Levophed and vasopressin drips were started, and IV fluids were continued to keep the systolic blood pressure above 100 to ensure adequate renal perfusion. Metronidazole, azithromycin, cefepime, and vancomycin were administered to cover septic shock from an unknown source. All sources pointed to a septic picture but no source could be found.

 As the patient was too unstable for a CT scan at that time, an ultrasound of the abdomen was performed to evaluate for a source of sepsis; it yielded no significant findings. A few days later, the patient was stable enough for CT. The cervical and thoracic spine did not show any evidence of diskitis or osteomyelitis. CT scan of the chest demonstrated small bilateral pleural effusions with atelectasis and atelectatic opacities, as well as left ventricular and pulmonary arterial enlargement. CT of the abdomen and pelvis revealed diffuse hepatic steatosis with some nonspecific pelvic free fluid and pelvic sidewall fat stranding. 

With all other sources of infection investigated a lumbar puncture was performed, which revealed gram-positive cocci in pairs as well as a significant white blood cell count. Blood cultures also grew gram-positive cocci in pairs, and a tracheal aspirate revealed Methicillin-resistant *Staphylococcus aureus* (MRSA). The patient was then started on vancomycin (rifampin was added at a later time following resolution of elevated liver function tests) for four weeks total to treat later confirmed *Staphylococcal meningitis* due to bacteremia from pneumonia. Blood cultures became negative 5 days into the patient's admission, and CSF culture one week after the patient's initial presentation was negative. A transesophageal echocardiogram one week after admission demonstrated a normalized ejection fraction and with no evidence of vegetations. 

Nearly two weeks into the patient's stay, he was extubated. However, his SpO2 dropped to nearly 40%, and the patient suffered another PEA arrest lasting two minutes and was reintubated. Soon after, a left chest tube was placed for a large pleural effusion that developed secondary to pneumonia; 1.6 liters of serosanguinous fluid was collected. The chest tube was removed 1 week later. 

The patient was extubated again eighteen days after his initial presentation. He tolerated room air sitting up with SpO2 98%; however, he dropped to 78–86% on room air while asleep, which was likely secondary to obstructive sleep apnea. Sleep medicine saw and evaluated the patient with polysomnography, and the patient was started on BiPAP at night. 

The patient remained hyponatremic throughout most of his stay; he was eventually diagnosed with the syndrome of inappropriate antidiuretic hormone secretion (SIADH) and treated with water restriction. It was near normal—134 mm/L—by the time he left the hospital. Finally, after a 38-day stay in the hospital, the patient was well enough for discharge. As the patient had become severely deconditioned during his time in the hospital, he was discharged to a subacute rehabilitation facility for further recovery with a final diagnosis of sepsis secondary to bacterial meningitis.

## 3. Discussion

While the initial vital signs were normal, the worsening vital signs showed markers of systemic inflammatory responses syndrome (SIRS) that may have been one of the first nonspecific indicators of an infectious process. The positive SIRS criteria were pulse of 128 beats per minute, respiration rate of 22 breaths per minute, and bands of 23%. Criteria for severe sepsis would have been met as the patient has a lactic acid of 3.6, however, no site of infection was ascertained until further in this patient's course. 

If bacterial meningitis is clinically apparent empiric antibiotics should be started. Current recommendations are for vancomycin to be started to cover increasing resistance to *S*. *pneumoniae* as well as a 3rd or 4th generation cephalosporin or chloramphenicol [1] for all adults with suspected antibiotics. These antibiotics cover for the common causes of bacterial menigitis in adults: *S. Pneumonia* and *N. Meningitidis*. While later in this patient's course it was found that he had previously been a cocaine abuser, it is unknown if he was ever an intravenous drug abuser. If it were known that he was an intravenous drug abuser, he would have been at risk for *Candida albicans* and *S. Aureus* as well as other organisms [2]. This may have altered our management and choice of antibiotics. Additionally, with the suspicion of severe sepsis the patient would have initially benefited from early goal-directed therapy targeting sepsis. 

 Classically it is taught that meningitis presents with the triad of fever, stiff neck, and altered mental status. While this may occur, a 2008 study showed that this classic triad is only present less than half of the cases of bacterial meningitis [3]. One prospective study found that 95% of patients with bacterial meningitis had at least 2 of the following: fever, headache, neck stiffness, and altered mental status [4]. This patient's diagnosis was complicated by the fact that he initially did not have the triad or headache; however later in his course, he did develop altered mental status and fever. Additionally the patient did not display the classic Kernig's or Brudzinsky's sign. The patient's diagnosis of meningitis was also complicated by an acute NSAID overdose and PEA arrest.

 Our patient admitted to taking approximately seventy 500 mg tablets of Ibuprofen. This was confirmed by sending the patient's sister to his apartment with the explicit instruction to return with all of the pill bottles in the apartment. The sister returned with an empty pill bottle missing seventy 500 mg ibuprofen tablets. The patient states that he had bought the bottle following his injury and had increased the dosage until he came to the emergency department when he ran out. The patient had had exceeded the maximum recommended dose of 2400 mg/day (or 10 mg/kg every 6 hours) by ingesting approximately 35,000 mg over 4 days. The patient was unable to state exactly how many pills he ingested over each day but stated that the majority were consumed over the last day as his pain increased. 

 Ibuprofen belongs to the nonsteroidal anti-inflammatory drug (NSAID) class, commonly used as an analgesic, anti-inflammatory, or antipyretic agent. This class of drug works by blocking cyclooxygenases (COX) conversion of arachidonic acid to endoperoxides. Endoperoxides are used to generate prostaglandin E2 (PGE2) that sensitize nociceptors to excitatory agents [5]. While NSAIDs have pain mitigation effects their reversible inhibition of the cyclooxygenase pathway affects, many additional organ systems. Morbidity and death from renal insufficiency and GI bleeding with chronic NSAID use far overshadow those of acute NSAID overdose [6].

 While being over-the-counter makes this drug very accessible, fatal exposures of NSAIDs are rare. In fact according to the American Association of Poison Control Centers from 1997 to 2000 there were only 35 deaths from isolated NSAID overdoses [7]. In a review of over 5000 NSAID overdoses, the most common signs and symptoms were nausea, vomiting, drowsiness, blurred vision, and dizziness. Less than 0.5 percent of these patients experienced severe harm (e.g., renal failure); most required no medical intervention [6]. Symptoms are unlikely after ingestion of 100 mg/kg or less and are usually not life-threatening unless more than 400 mg/kg is ingested [8]. 

Our patient ingested approximately 300 mg/kg. As the time since last ingestion of Ibuprofen was unknown, initial decontamination was attempted with 50 g of activated charcoal. This practice is in line with the current Joint Position Statement of the American Academy of Clinical Toxicology and the European Association of Poisons Centers and Clinical Toxicologists and recommends that activated charcoal should be administered to patients who present within 1 hour of ingestion of a significant overdose, in an attempt to reduce absorption [6]. This is because NSAIDs are rapidly absorbed following oral ingestion, with peak concentrations occurring within 2 hours of ingestion of nonsustained release preparations, and symptoms of massive ingestion usually occur within 4 hours [9]. 

Consistent with many case reports our patient too had an increased anion-gap metabolic acidosis. With NSAID overdoses this usually occurs as a result of accumulation of toxic metabolites (the patterns of toxicity and management of acute nonsteroidal anti-inflammatory drug (NSAID) overdose). In addition to the anion gap metabolic acidosis our patient developed renal failure due to his NSAID overdose. In patients with normal renal physiology, prostaglandins are responsible for the dilatation of the afferent arteriole of the glomerulus. This dilatation normally serves to increase renal plasma flow and increase glomerular filtration fraction. When the production of prostaglandins is blocked by NSAIDS, the afferent arteriole is blocked and dilatation cannot occur leading to decreased glomerular filtration rate. 

## Figures and Tables

**Table 1:** Initial labs.

Urine Drug Screen	
Opiates—Positive	
Cocaine—Negative	
Amphetamine—Negative	
Cannabinoids—Negative	
Barbituates—Negative	
Benzodiazepines—Positive	
CBC	
WBC Count 7.6 K/uL	
RBC Count 3.96 M/uL	
Hemoglobin 12.2 g/dL	
Hematocrit 36.0%	
MCV 90.8 fl	
MCH 30.7 pg	
MCHC 33.8 g/dL	
RDW 12.7%	
Platlet Count 158 K/uL	
Differential	
Neutorphil 60%	
Lymphocyte 4%	
Monocyte 9%	
Eosinophil 0%	
Basophil 0%	
Neutorphil Absolute 4.57 K/uL	
Lymphocyte Absolute 0.30 K/uL	
Monocyte Absolute 0.68 K/uL	
Eosinophil Absolute 0.00 K/uL	
Basophil Absolute 0.00 K/uL	
Band 23%	
Metamyelocyte 4%	
Band Absolute 1.75 K/uL	
Metamyelocyte Absolute 0.30	
RBC morphology normal	
Urinalysis	
Type—Clean Catch	
Color—Amber	
Clarity—Cloudy	
Specific Gravity—1.025	
pH 5.5	
Protein 100 mg/dL	
Glucose 100 mg/dL	
Ketones—Trace	
Bilirubin—Negative	
Blood—Moderate	
Urobilinogen—1.0 U/dL	
Nitrite—Negative	
Leukocyte Esterase—Negative	
Urinalysis Micro	
RBC 0 to 2/HPF	
WBC 5 to 10/HPF	
Bacturia Moderate	
Arterial Blood Gas	
pH 7.52	
PCO_2_22.0 mmHg	
PO_2_ 194.6	
Base Deficit 3.1 mmol/L	
HCO_3_ 17.5 mmol/L	
TCO_2_ 18.2 mmol/L	
Oxygen Saturation 99.3	
Gas—Room Air	
Glucose 210	
ALT/SGPT 39 IU/L	
Alkaline Phosphatase 90 IU/L	
Bilirubin ?	
AST/SGOT 43 IU/L	
Lactic Acid 3.6 mmol/L	
Troponin I 0.04 ng/mL	
Electrolytes	
Sodium 122 mmol/L	
Potassium 3.2 mmol/L	
Chloride 84 mmol/L	
Carbon Dioxide 23 mmol/L	
Anion Gap 15	
Magnesium 2.5 mg/dL	
Phosphorus 1.7 mg/dL	
